# A Randomized, Placebo-Controlled, Respiratory Syncytial Virus Human Challenge Study of the Antiviral Efficacy, Safety, and Pharmacokinetics of RV521, an Inhibitor of the RSV-F Protein

**DOI:** 10.1128/AAC.01884-19

**Published:** 2020-01-27

**Authors:** John DeVincenzo, Dereck Tait, John Efthimiou, Julie Mori, Young-In Kim, Elaine Thomas, Lynn Wilson, Rachel Harland, Neil Mathews, Stuart Cockerill, Kenneth Powell, Edward Littler

**Affiliations:** a University of Tennessee Center for Health Sciences, Memphis, Tennessee, USA; b Children’s Foundation Research Institute at LeBonheur Children’s Hospital, Memphis, Tennessee, USA; c ReViral Ltd., Stevenage, Hertfordshire, United Kingdom; d Independent Respiratory Specialist, Oxford, United Kingdom; e hVIVO Services Limited, London, United Kingdom

**Keywords:** antiviral, respiratory syncytial virus, RV521

## Abstract

Effective treatments for respiratory syncytial virus (RSV) infection are lacking. Here, we report a human proof-of-concept study for RV521, a small-molecule antiviral inhibitor of the RSV-F protein. In this randomized, double-blind, placebo-controlled trial, healthy adults were challenged with RSV-A Memphis-37b. After infection was confirmed (or 5 days after challenge virus inoculation), subjects received RV521 (350 mg or 200 mg) or placebo orally every 12 h for 5 days. The primary endpoint was area under the curve (AUC) for viral load, as assessed by reverse transcriptase quantitative PCR (RT-qPCR) of nasal wash samples. The primary efficacy analysis set included subjects successfully infected with RSV who received ≥1 dose of study drug. A total of 66 subjects were enrolled (*n *= 22 per group); 53 were included in the primary analysis set (RV521 350 mg: *n *= 16; 200 mg: *n *= 18; placebo: *n *= 19). The mean AUC of RT-qPCR-assessed RSV viral load (log_10_ PFU equivalents [PFUe]/ml · h) was significantly lower with RV521 350 mg (185.26; standard error [SE], 31.17; *P = *0.002) and 200 mg (224.35; SE, 37.60; *P = *0.007) versus placebo (501.39; SE, 86.57). Disease severity improved with RV521 350 mg and 200 mg versus placebo (*P = *0.002 and *P = *0.009, respectively, for AUC total symptom score [score × hours]). Daily nasal mucus weight was significantly reduced (*P = *0.010 and *P = *0.038 for RV521 350 mg and 200 mg, respectively, versus placebo). All treatment-emergent adverse events were grade 1 or 2. No subjects discontinued due to adverse events. There was no evidence of clinically significant viral resistance, and only three variants were detected. RV521 effectively reduced RSV viral load and disease severity in humans and was well tolerated. (This study has been registered at ClinicalTrials.gov under registration no. NCT03258502.)

## Abstract (precis)

Effective treatments for respiratory syncytial virus (RSV) infection are lacking. Here, we report a human proof-of-concept study for RV521, a small-molecule antiviral inhibitor of the RSV-F protein. In this randomized, double-blind, placebo-controlled trial, healthy adults were challenged with RSV-A Memphis-37b.

## INTRODUCTION

Respiratory syncytial virus (RSV) is a common cause of respiratory infections across all age groups but has the most severe impact in young children and vulnerable adult populations, including the elderly, the immunocompromised, and those with chronic obstructive pulmonary disease (1–3). In young children with acute lower respiratory tract infections (LRTIs), RSV is the most commonly identified pathogen, causing significant morbidity and mortality (3, 4). In those aged <1 year, RSV causes approximately 15 times as many hospitalizations (5) and nearly 10 times as many estimated annual respiratory deaths as influenza (6). Worldwide in 2015, RSV was responsible for an estimated 2.7 to 3.8 million hospital admissions and 48,000 to 74,500 in-hospital deaths among children under 5 years of age (4). RSV-infected infants are also at increased risk of developing asthma later in childhood or during adolescence (7, 8). In elderly and high-risk adult populations, the disease burden of RSV is comparable to that of influenza (1, 9).

No vaccines are currently available to prevent RSV infection. Palivizumab, an RSV monoclonal antibody, is approved for the prevention of serious RSV LRTI but has limited efficacy and a high cost (10, 11). Furthermore, use of palivizumab is restricted to high-risk infants, including those born prematurely or with underlying conditions, who comprise <3% of the birth cohort (12, 13). An aerosolized formulation of the antiviral agent ribavirin is approved in the United States for the treatment of severe LRTIs caused by RSV in hospitalized infants and young children (14). However, it has demonstrated limited antiviral potency *in vitro* (15) and is rarely used in clinical practice due to lack of clinical benefit and concerns regarding toxicity (including bone marrow suppression and potential oncogenic and teratogenic activity) (11, 14).

RV521 is an orally available small-molecule inhibitor of the RSV-F protein (Fig. S1) that has exhibited potent efficacy against a panel of clinical isolates of RSV-A and RSV-B viruses *in vitro* (50% inhibitory concentration [IC_50_] [range], 1.4 nM [0.3 to 10.4] for RSV-A clinical isolates [*n* = 20] and 1.0 nM [0.1 to 2.1] for RSV-B isolates [*n* = 16]) (16). RV521 has demonstrated a good safety profile in preclinical evaluations, including juvenile toxicology studies (17). In a first-in-human, single- and multiple-ascending dose study conducted in 76 healthy adult males, RV521 was well tolerated, with no discontinuations due to adverse events, and it displayed a favorable pharmacokinetic (PK) profile (17).

We therefore performed a phase 2a, double-blind, placebo-controlled, clinical study to establish human therapeutic proof-of-concept for the antiviral activity of RV521 in the treatment of an established RSV infection, using a virus challenge model per regulatory guidance (14, 18). Also, the safety, tolerability, and PK profile of RV521 were assessed, and viral RSV fusion (RSV-F) gene sequence analysis was performed to detect any viral variants following RV521 treatment.

## RESULTS

**FIG 1:**
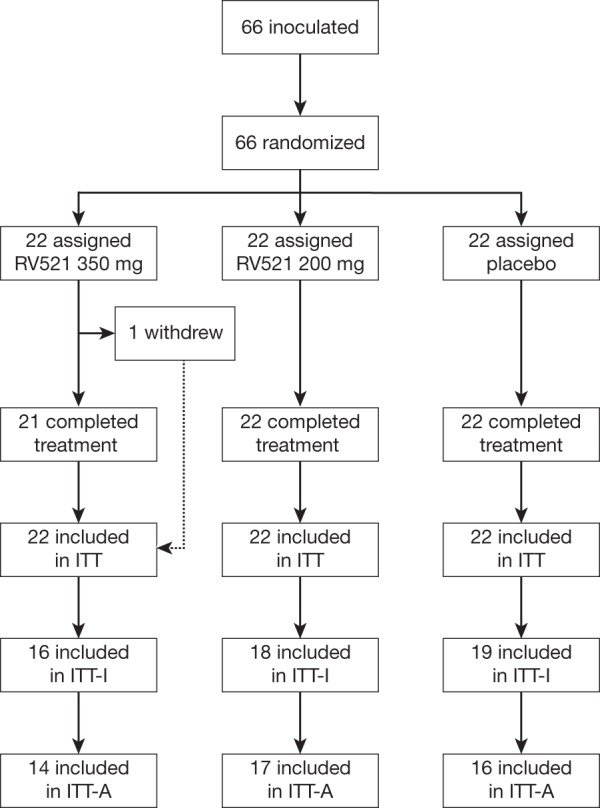
Subject disposition. The ITT analysis set included all randomized subjects who received the challenge virus and at least one dose of study drug. The ITT-I analysis set included all randomized subjects who received challenge virus and at least one dose of study drug and met the criterion for laboratory-confirmed RSV infection (presence of viral shedding). The ITT-A analysis set was a subset of the ITT-I population that included subjects in whom RSV infection was confirmed before administration of study drug. One subject assigned to RV521 350 mg withdrew consent following three doses. ITT, intent-to-treat; ITT-I, intent-to-treat infected; ITT-A, intent-to-treat infected A; RSV, respiratory syncytial virus.

In total, 66 subjects were recruited between 27 July 2017 and 28 September 2017. All were inoculated with RSV challenge virus (RSV-A Memphis-37b) and randomly assigned to placebo or RV521 groups in a 1:2 placebo:RV521 design (Fig. 1). In cohort 1, 33 subjects received RV521 (*n *= 22) or placebo (*n *= 11) dosed at 200 mg. In cohort 2, 33 subjects received RV521 (*n *= 22) or placebo (*n *= 11) dosed at 350 mg. One subject (an RV521 350 mg recipient) withdrew consent for reasons unrelated to treatment after receiving three of 10 planned doses. This subject returned for the day 28 visit but, not fulfilling intent-to-treat infected (ITT-I) criteria, was excluded from this primary efficacy analysis set. Therefore, 32 subjects in cohort 2 were treated according to the protocol-defined dosing regimen. Table 1 shows subject baseline characteristics; no differences were observed across treatment groups.

**TABLE 1:** Baseline subject characteristics

Characteristic	Treatment group		
	RV521 350 mg (*N* = 22)	RV521 200 mg (*N* = 22)	Placebo (*N* = 22)
Male, *n* (%)	16 (73)	13 (59)	15 (68)
Ethnicity, *n* (%)			
Caucasian	18 (82)	21 (95)	21 (95)
South Indian	0	0	1 (5)
Other	4 (18)	1 (5)	0
Age (yrs)			
Mean (SD)	24.5 (5.50)	21.7 (3.09)	24.6 (5.29)
Range	18–40	19–34	19–39
Height (cm)			
Mean (SD)	175.24 (8.22)	172.83 (8.11)	176.50 (8.58)
Range	158.2–188.6	161.0–194.5	163.2–190.0
Weight (kg)			
Mean (SD)	72.75 (10.38)	70.48 (10.42)	75.25 (10.55)
Range	57.8–92.7	57.9–94.6	61.4–103.6
BMI^*a*^ (kg/m^2^)			
Mean (SD)	23.56 (2.24)	23.53 (2.69)	24.15 (2.60)
Range	20.0–28.2	19.4–28.1	19.4–29.6
Neutralizing antibody titer prior to RSV inoculation^*b*^			
Median	810	1107	810
Range	156–1403	270–4209	270–4209

a BMI, body mass index.

b RSV neutralizing antibody titers were measured during screening, and only subjects with a value ≤810 were enrolled. Titers were measured again prior to RSV inoculation, and these values are reported here.

**FIG 2:**
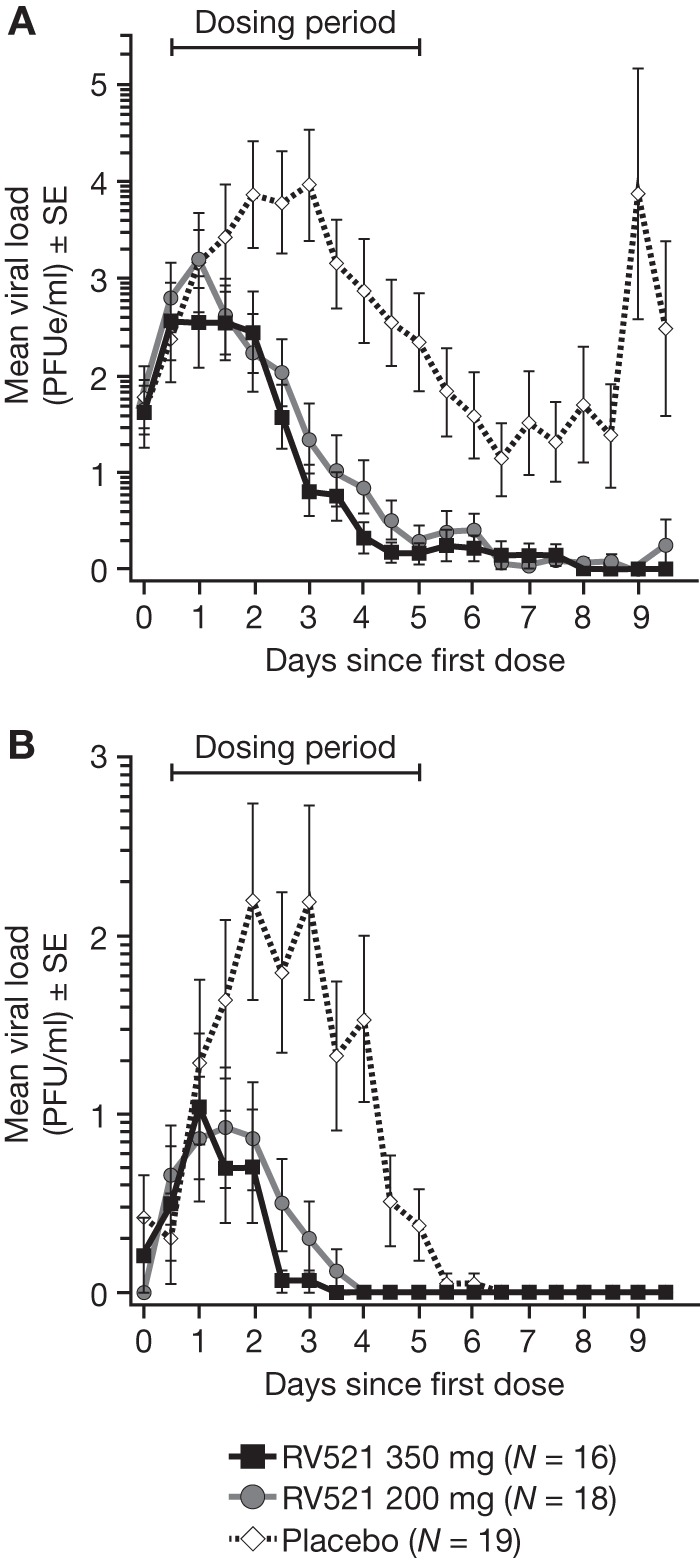
Mean viral load by nasal wash RT-qPCR (A) and by nasal wash quantitative culture (B) by day relative to dosing (ITT-I analysis set). Once RSV infection was confirmed (i.e., RSV RNA detected by qualitative integrated cycler PCR), subjects were assigned a randomization number; treatment was initiated 12 h (±1 h) after the confirmatory RSV-positive nasal wash sample had been collected. Viral load (RT-qPCR) appeared to rebound after day 8.5 in the placebo arm. However, this apparent increase resulted from the staggered randomization of subjects (the mean viral load at day 9 was calculated from just four subjects, three of whom had consistently high viral loads throughout the study). ITT-I, intent-to-treat infected (all randomized subjects who received the challenge virus and at least one dose of study drug and met the criterion for laboratory-confirmed RSV infection [presence of viral shedding]); PFUe, PFU equivalents; RSV, respiratory syncytial virus; RT-qPCR, reverse transcriptase quantitative PCR; SE, standard error.

Viral loads were consistently reduced with RV521. After achieving similar pretreatment baseline viral loads, significant differences were observed with RV521 recipients compared with those receiving placebo (ITT-I; Table 2). The magnitude and dynamics of the antiviral effects of RV521 are shown in Fig. 2. The primary endpoint (mean area under the curve [AUC] of viral load as assessed by reverse transcriptase quantitative PCR [RT-qPCR]; ITT-I) was significantly reduced in the RV521 350 mg (185.26 log_10_ PFU equivalents [PFUe]/ml · h [*P = *0.002]) and RV521 200 mg (224.35 log_10_ PFUe/ml · h [*P = *0.007]) groups compared with placebo (501.39 log_10_ PFUe/ml · h). The percentage reduction in mean AUC for RV521 350-mg and RV521 200-mg groups relative to that for the placebo group was 63.05% and 55.25%, respectively (98.87% and 99.10% reduction, respectively, when AUC values of unlogged RT-qPCR data were compared). There was no significant difference in RT-qPCR AUC between the 200-mg and 350-mg RV521 dose groups (*P = *0.429; Satterthwaite *t* test). A significant reduction in AUC of viral load as assessed by quantitative viral culture was also observed with RV521 versus placebo (percentage reduction in mean AUC for RV521 350-mg and RV521 200-mg groups relative to that of the placebo group was 76.42% [*P = *0.012] and 68.60% [*P = *0.027], respectively). Mean peak RT-qPCR-assessed viral load was significantly reduced with both doses of RV521 versus placebo (3.17, 3.47, and 4.77 log_10_ PFUe/ml for RV521 350 mg [*P = *0.024], RV521 200 mg [*P = *0.031], and placebo, respectively). Because peak viral load was lowered, RV521 treatment also significantly reduced the elapsed time until peak viral load occurred (time to peak viral loads was 1.63, 0.95, and 2.68 days for RV521 350 mg [*P = *0.024], RV521 200 mg [*P < *0.0001], and placebo, respectively). Mean peak, but not time to peak, viral load was significantly lower with both doses of RV521 versus placebo when assessed by quantitative culture (mean peak *P = *0.012 and *P = *0.016 for RV521 350 mg and 200 mg, respectively). At the time that peak viral load was occurring in the placebo group, mean RT-qPCR-assessed viral load was 3.16 and 2.61 log_10_ PFUe/ml lower with RV521 350 mg and 200 mg, respectively, compared with that with placebo, and mean quantitative culture-assessed viral load was 1.49 and 1.32 log_10_ PFU/ml lower with RV521 350 mg and 200 mg, respectively. The median duration of time to a viral load of <1 log_10_ as assessed by RT-qPCR was significantly shorter with RV521 350 mg (3.5 days; *P = *0.0001) and RV521 200 mg (3.0 days; *P = *0.0003) versus placebo (6.5 days). The median duration of time to undetectable viral load assessed using quantitative culture was significantly shorter in both RV521 groups versus the placebo group (*P < *0.0001 for both) (Table 2; see also Fig. S2).

**TABLE 2:** Viral load endpoints (ITT-I analysis set)

Parameter^*e*^	Treatment group		
	RV521 350 mg (*N* = 16)	RV521 200 mg (*N* = 18)	Placebo (*N* = 19)
AUC of viral load (RT-qPCR) (log_10_ PFUe/ml · h)			
Mean (SE)	185.26 (31.17)	224.35 (37.60)	501.39 (86.57)
Difference in mean relative to that of placebo (95% CI)	−316.14 (−506.71, −125.57)	−277.04 (−471.63, −82.46)	
Reduction in mean vs that of placebo (%)	63.05	55.25	
*P* value^*a*^	0.002	0.007	
AUC of viral load (RT-qPCR) (log_10_ PFUe/ml · h [fixed time period of 6.5 days])			
Mean (SE)	182.59 (30.29)	221.98 (37.05)	435.96 (65.12)
Reduction in mean vs that of placebo (95% CI)	−253.37 (−401.21, −105.53)	−213.98 (−367.35, −60.61)	
Reduction in mean vs placebo (%)	58.12	49.08	
*P* value^*a*^	0.002	0.008	
AUC of viral load (RT-qPCR, unlogged) (PFUe/ml · h)			
Mean (SE)	1,356,521.31 (1,117,131.13)	1,087,294.53 (604,070.11)	120,190,244.05 (50,659,684.44)
Reduction in mean vs that of placebo (%)	98.87	99.10	
*P* value^*b*^	0.023	0.019	
AUC of viral load (quantitative culture) (log_10_ PFU/ml · h)			
Mean (SE)	38.29 (13.36)	50.98 (14.89)	162.35 (37.77)
Reduction in mean vs that of placebo (%)	76.42	68.60	
*P* value^*b*^	0.012	0.027	
Pretreatment viral load (RT-qPCR) (log_10_ PFUe/ml)			
Mean (SE)	1.60 (0.34)	1.64 (0.26)	1.77 (0.32)
Peak viral load (RT-qPCR) (log_10_ PFUe/ml)			
Mean (SE)	3.17 (0.45)	3.47 (0.30)	4.77 (0.49)
Difference in mean relative to that of placebo (95% CI)	−1.59 (−2.96, −0.23)	−1.30 (−2.47, −0.13)	
*P* value^*c*^	0.024	0.031	
Peak viral load (quantitative culture) (log_10_ PFU/ml)			
Mean (SE)	1.58 (0.41)	1.72 (0.40)	3.255 (0.46)
Difference in mean relative to that of placebo (95% CI)	−1.67 (−2.95, −0.40)	−1.54 (−2.77, −0.30)	
*P* value^*c*^	0.012	0.016	
Time to peak viral load (RT-qPCR) (days)			
Mean (SE)	1.63 (0.34)	0.95 (0.11)	2.68 (0.28)
Difference in mean relative to that of placebo (95% CI)	−1.06 (−1.96, −0.15)	−1.74 (−2.36, −1.11)	
*P* value^*a*^	0.024	<0.0001	
Time to peak viral load (quantitative culture) (days)			
Mean (SE)	3.85 (0.80)	3.69 (0.81)	3.34 (0.50)
*P* value^*b*^	0.895	0.447	
Mean viral load at time of peak viral load in placebo arm (day 3; RT-qPCR) (log_10_ PFUe/ml)			
Mean (SE)	0.80 (1.07)	1.35 (0.36)	3.96 (0.57)
Reduction in mean vs placebo, log_10_ PFUe/ml (%)	3.16 (79.71)	2.61 (65.96)	
Mean viral load at time of peak viral load in placebo arm (day 2; quantitative culture) (log_10_ PFU/ml)			
Mean (SE)	0.71 (0.32)	0.88 (0.30)	2.20 (0.55)
Reduction in mean vs that of placebo, log_10_ PFU/ml (%)	1.49 (67.90)	1.32 (60.10)	
Time to <1 log_10_ viral load, (RT-qPCR) (days)			
Median (Q1, Q3)	3.5 (3.0, 4.0) (*n* = 13)	3.0 (3.0, 6.0) (*n* = 17)	6.5 (5.5, 8.5) (*n* = 17)
*P* value^*d*^	0.0001	0.0003	
Time to undetectable viral load, (quantitative culture) (days)			
Median (Q1, Q3)	2.5 (2.0, 2.5) (*n* = 9)	3.0 (2.0, 3.5) (*n* = 11)	4.5 (4.0, 5.5) (*n* = 16)
*P* value^*d*^	<0.0001	<0.0001	

a Satterthwaite *t* test.

b Wilcoxon rank-sum test.

c *t* test.

d Kaplan-Meier log-rank test.

e AUC, area under the curve; CI, confidence interval; ITT-I, intent-to-treat infected (defined as all randomized subjects who received the challenge virus and at least one dose of study drug and met the criterion for laboratory-confirmed RSV infection [presence of viral shedding]); PFUe, PFU equivalents; Q1, Q3, interquartile range; RSV, respiratory syncytial virus; RT-qPCR, reverse transcriptase quantitative PCR; SE, standard error.

**FIG 3:**
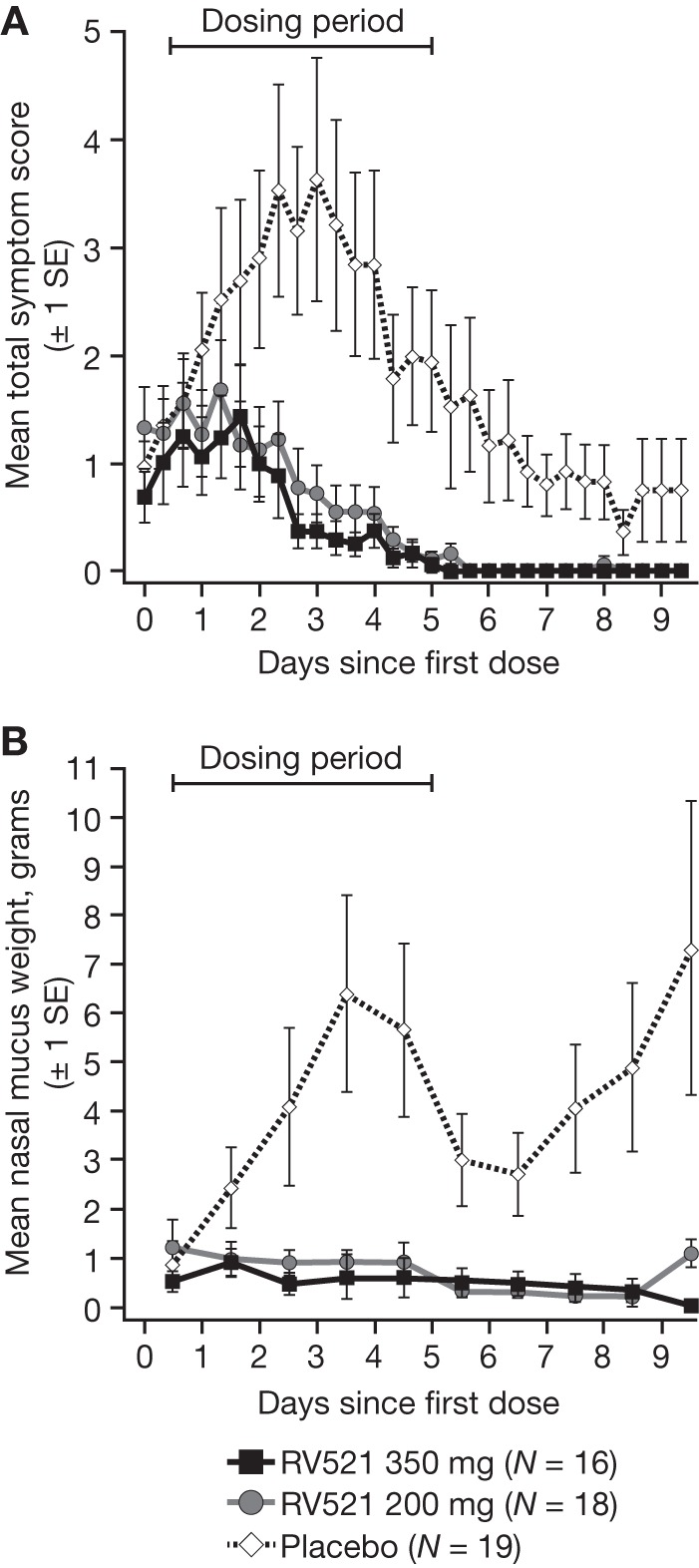
Mean total symptom score (10-item symptom diary card) (A) and mean total nasal mucus weight (B) by day relative to dosing (ITT-I analysis set). Once RSV infection was confirmed (i.e., RSV RNA detected by qualitative integrated cycler PCR), subjects were assigned a randomization number; treatment was initiated 12 h (±1 h) after the confirmatory nasal wash sample had been taken. The apparent late increase in mucus weight observed in the placebo arm was due to the staggered randomization of subjects. ITT-I, intent-to-treat infected (all randomized subjects who received the challenge virus and at least one dose of study drug and met the criterion for laboratory-confirmed RSV infection [presence of viral shedding]); RSV, respiratory syncytial virus; SE, standard error.

Disease severity due to RSV infection was consistently reduced with RV521 compared to that with placebo (Table 3; Fig. 3). In the ITT-I analysis set, RV521 350-mg and 200-mg doses significantly reduced AUC total symptom scores (percentage reduction relative to placebo, 78.42% [*P = *0.002] and 70.84% [*P = *0.009], respectively). Both doses of RV521 also significantly reduced the peak total symptom score versus that of the placebo group (1.9 [*P = *0.016] and 2.3 [*P = *0.034] for RV521 350 mg and 200 mg, respectively, versus 5.1 with placebo). Nasal mucus weight data were not normally distributed, necessitating *post hoc* analysis of this endpoint. Least-squares (LS) mean daily nasal mucus weight was significantly lower with RV521 350 mg and 200 mg versus that with placebo (0.27 g [*P = *0.010] and 0.33 g [*P = *0.038], respectively, versus 0.61 g). Results of the sensitivity analyses (based on the intent-to-treat infected A [ITT-A] analysis set and a fixed 6.5-day period) did not differ markedly from those of the main analyses with respect to antiviral efficacy and RSV disease-reducing effect (Table 2; see also Tables S1 to S3).

**TABLE 3:** Disease severity endpoints (ITT-I analysis set)

Parameter^*d*^	Treatment group		
	RV521 350 mg (*N* = 16)	RV521 200 mg (*N* = 18)	Placebo (*N* = 19)
AUC total symptom score (score × hours)			
Mean (SE)	82.41 (24.45)	111.35 (33.88)	381.82 (111.59)
Reduction in mean relative to that of placebo (%)	78.42	70.84	
*P* value^*a*^	0.002	0.009	
Peak total symptom score			
Mean (SE)	1.9 (0.45)	2.3 (0.48)	5.1 (1.11)
Difference in mean relative to that of placebo (95% CI)	−3.12 (−5.59, −0.64)	−2.72 (−5.22, −0.22)	
*P* value^*b*^	0.016	0.034	
Time to peak total symptom score (days)			
Mean (SE)	1.56 (0.57)	2.08 (0.68)	1.83 (0.23)
Difference in mean relative to that of placebo (95% CI)	−0.26 (−1.56, 1.03)	0.25 (−1.24, 1.75)	
*P* value^*b*^	0.675	0.731	
Daily nasal mucus weight (g)			
LS mean^*c*^	0.27	0.33	0.61
Difference in LS mean relative to that of placebo (%)	55.74	45.90	
*P* value^*a*^	0.010	0.038	

a Wilcoxon rank-sum test.

b Satterthwaite *t* test.

c LS mean was calculated from a mixed model with repeated measures, adjusted for baseline mucus weight and treatment group as covariates and subject as a random effect. The *P* value represents the LS mean difference between treatment groups.

d AUC, area under the curve; CI, confidence interval; ITT-I, intent-to-treat infected (defined as all randomized subjects who received the challenge virus and at least one dose of study drug and met the criterion for laboratory-confirmed RSV infection [presence of viral shedding]); LS, least squares; RSV, respiratory syncytial virus; SE, standard error.

Adverse events (AEs) occurred in 45 of 66 (68%) subjects (both treated and placebo groups). Fourteen AEs in 12 subjects were reported after inoculation with challenge virus but before administration of study treatment. Eighty-five AEs (37 subjects) were treatment emergent (23 in 11 subjects in the placebo group, 19 in 11 subjects in the RV521 200-mg group, and 43 in 10 subjects in the RV521 350-mg group). No treatment-related serious adverse events (SAEs), deaths, or discontinuations due to AEs occurred in any treatment group. One SAE (acute myocarditis) was reported. An elevated troponin level was detected during a scheduled laboratory safety evaluation in an asymptomatic placebo recipient. Further evaluation revealed a normal electrocardiogram (ECG) and a cardiac scan interpreted as consistent with mild myocarditis. The event resolved spontaneously. Fifteen treatment-emergent adverse events (TEAEs) (10 subjects) were grade 2 in severity (9 events in 5 subjects in the combined placebo group and 2 in 2 subjects and 4 in 3 subjects in the RV521 200-mg and 350-mg groups, respectively); there were no TEAEs of grade ≥3. The majority of TEAEs were grade 1 gastrointestinal events (nausea and diarrhea), which occurred more frequently in the RV521 350-mg dose group than in the RV521 200-mg dose group (59% versus 32% of subjects, respectively). In general, the incidence of these events did not increase in subjects during the dosing period, were transient, and resolved without medication. One subject in the RV521 350-mg group reported grade 2 diarrhea and abdominal pain following discharge from the unit (onset approximately 4 days after the last dose of RV521) and self-medicated with a single dose of loperamide hydrochloride and hyoscine butylbromide. Table S4 lists all TEAEs; those that occurred in ≥2 subjects in any treatment group are included in Table 4.

**TABLE 4:** Treatment-emergent adverse events that occurred in ≥2 subjects in any treatment group (safety analysis set)^*a*^

TEAE^*b*^	No. of subjects (%) for treatment group:		
	RV521 350 mg (*N* = 22)	RV521 200 mg (*N* = 22)	Placebo (*N* = 22)
Abdominal pain	5 (23)	2 (9)	0
Diarrhea	9 (41)	3 (14)	1 (5)
Nausea	12 (55)	2 (9)	2 (9)
Vomiting	2 (9)	1 (5)	0
Rhinitis	2 (9)	1 (5)	1 (5)
URTI	0	2 (9)	0
Viral URTI	2 (9)	0	0
Headache	0	0	2 (9)
Rash	0	0	2 (9)

a Respiratory tract infection symptoms were only captured as an AE if they were unexpected as a result of the virus challenge, met the criteria for an AE, and were deemed clinically significant in the opinion of the investigator.

b AE, adverse event; TEAE, treatment-emergent adverse event; URTI, upper respiratory tract infection.

No notable differences in clinical or laboratory tests, such as vital signs or liver enzymes, between RV521- and placebo-treated subjects were observed, and there were no clinically significant ECG findings. Spirometry showed that one subject (RV521 350-mg group) had intermittent drops in forced expiratory volume in 1 s (FEV_1_) of >15%, which began after inoculation with challenge virus but before commencement of study drug; this was considered clinically significant and possibly related to the challenge virus.

Mean maximum RV521 plasma concentration following a single dose (dose 1) and repeated twice-daily dosing (dose 10) at 200 mg and 350 mg, respectively, was 55.3 ng/ml and 169 ng/ml after dose 1, and 94.9 ng/ml and 294 ng/ml after dose 10. The median time to maximum plasma concentration was 5 to 6 h postdose. Target trough levels (3× protein-adjusted *in vitro* 90% effective concentration [EC_90_]) were achieved for 50% and 73% of subjects treated with RV521 200 mg after the first and second dose, respectively, and trough levels in excess of the target were achieved after the first dose for all subjects treated with RV521 350 mg. Steady-state plasma levels appeared to be reached by 24 h after the first dose of RV521 (200 mg and 350 mg), consistent with an elimination half-life of approximately 6 h. Following single (dose 1) and repeated twice-daily dosing (dose 10), systemic exposure increased with increasing RV521 dose at a greater than dose-proportional rate. The extent of accumulation of RV521 following repeated dosing at either dose level was consistent with linear kinetic theory. Mean PK parameters of RV521 following single and repeated twice-daily dosing are shown in Table S5.

Sequence analysis of the entire RSV-F gene was performed on nasal wash samples obtained from all 53 subjects in the ITT-I analysis set. The following three variants (amino acid position) were detected: G453D (present in one sample 6 days post first dose in a subject treated with RV521 200 mg), L141F (present in one sample 6 days post first dose in a subject treated with RV521 200 mg), and P389L (present in two samples, at 1.5 days and 5 days post first dose, in a subject treated with RV521 350 mg). No rebound in viral load or symptoms was observed after detection of any of these variants. No amino acid changes were detected in the RSV-F gene in samples from placebo-treated subjects. No viable RSV was quantifiable from any of the samples in which RSV-F protein variants were identified.

## DISCUSSION

The primary endpoint of this virus challenge study was met, with RV521 treatment at both 200 mg and 350 mg resulting in a statistically significant reduction in AUC of RT-qPCR-assessed RSV viral load relative to that with placebo. RV521 treatment also led to statistically significant improvements in multiple secondary viral load endpoints.

Other compounds that have been tested using this RSV challenge model include the oral nucleoside analogue prodrug ALS-008176 (19) and two inhibitors of the RSV-F protein, GS-5806 (12) and JNJ-53718678 (20). Observed reductions in RT-qPCR-assessed AUC viral load relative to that with placebo in these studies were 73 to 88% with ALS-008176 (19), 38 to 67% with GS-5806 (22 to 77% as assessed by quantitative culture) (12), and 41 to 53% with JNJ-53718678 (9 to 47% as assessed by quantitative culture) (20). Although not directly comparable, the results arising from these different studies, which used the same or very similar RSV challenge study designs, show that the magnitude of viral load reduction by RV521 treatment compares favorably with ALS-008176 and suggests an improvement over JNJ-53718678 and the majority of the GS-5806 dosing regimens tested. While ALS-008176 and GS-5806 demonstrated positive results in challenge studies, development of ALS-008176 has since been suspended and GS-5806, which was only evaluated in adult populations, failed to significantly reduce the viral load or improve clinical outcomes in hospitalized RSV-infected adults treated relatively late in their disease course (21).

Safety and tolerability data observed with RV521 in the current study were favorable and consistent with phase 1 data (unpublished). AEs were generally graded 1 in severity and transient in nature. There were no treatment-related SAEs and no subject discontinuations due to AEs. While gastrointestinal TEAEs occurred more frequently with RV521 than with placebo, the majority of these events were transient, mild, resolved without concomitant medication, and did not lead to discontinuation in any individual. Although cross-study comparisons of data are inherently limited, the observed safety profile of RV521 seems to compare favorably with that of other anti-RSV agents (19, 20).

PK data from the current study suggest that RV521 200 mg and 350 mg are effective therapeutic doses in adult subjects, both resulting in significant improvements compared with placebo in primary and secondary viral load and disease severity endpoints. No significant differences in RT-qPCR AUC were observed between the 200-mg and 350-mg dose groups, although it should be noted that the study was not designed to assess differences in treatment effect between the two doses. The terminal half-life of RV521 (8.54 to 9.35 h) is shorter than or comparable to that reported for other studied anti-RSV compounds (33 to 35 h for GS-5806 [12], 63 h for ALS-008176 [19], and 6.5 to 10.5 h for JNJ-53718678 [20]), which may offer advantages, especially in relation to pediatric dosing, in avoiding accumulation and potential toxicity. The safety and PK of RV521 will be assessed in infants hospitalized with RSV infection in a planned phase 2a study.

There was no evidence of clinical resistance, with only three RSV-F genetic variants detected (G453D, P389L, and L141F), and no evidence of viral rebound or prolongation of clinical symptoms of RSV was observed in the subjects from whom these samples were taken. L141F is known to confer resistance to inhibitors of the RSV-F protein *in vitro* (22), although viruses mutated at this point have also been shown to have reduced fitness compared to that of the wild type (23). The detected G453D and P389L RSV-F protein variants have not been reported to reduce susceptibility to inhibitors of the RSV-F protein. However, P389L was detected in a placebo-treated RSV-A Memphis-37b-infected subject in another virus challenge study (personal communication, Y. H. Grad) (24), and therefore its occurrence in our study most likely resulted from natural variation rather than from RV521 treatment. Findings from the mutation analysis conducted during our clinical study compare favorably with those reported for GS-5806, in which treatment-emergent mutations conferring reduced susceptibility *in vitro* to GS-5806 were detected in 14 of 87 subjects treated with the agent and challenged with RSV (and in 0 of 53 placebo-treated subjects) (22). In the virus challenge study of ALS-008176, no mutations known to be associated with *in vitro* resistance to ALS-008176 were detected, although the study sample size was relatively small (29 subjects, with 17 receiving ALS-008176 and 12 receiving a placebo) (19). The low frequency of detected mutations following RV521 treatment suggested in our study is encouraging with respect to the treatment of pediatric patients and immunocompromised individuals, who have a greater potential for resistance to develop due to higher viral loads and longer durations of viral shedding than those in immunocompetent adults (19, 25). Characterization of the detected variants introduced synthetically into the RSV-F protein is the focus of ongoing studies.

Use of an RSV challenge model is recommended by regulatory authorities and allows potential RSV treatments to be critically evaluated in healthy adults while avoiding undue risk to vulnerable patient groups (14). Of note, AK0529, an inhibitor of the RSV-F protein, is being assessed in a phase 2 study (ClinicalTrials.gov identifier NCT02654171) in infants hospitalized with RSV infection, without having undergone prior testing in a challenge model study. The specific virus challenge model used in this study (experimental infection with RSV-A Memphis-37b) features aspects that reflect natural infection (26, 27) and involves commencing treatment after infection has been confirmed and after symptoms have started, as in a clinical setting. However, differences between the virus challenge model and natural infection mean that extrapolation of findings should be performed with caution. For example, virus challenge models largely result in upper respiratory tract infection, rather than in the more serious LRTI seen in naturally infected individuals. Naturally occurring LRTI may progress to severe lung disease in vulnerable populations, which would be targeted by effective anti-RSV agents. Furthermore, enrolled subjects are immunocompetent, and thus differ from some potential adult populations (e.g., the immunocompromised or elderly). Also, treatment in our virus challenge model study was typically administered 12 h after confirmation of RSV infection, significantly earlier than in individuals with natural infection, who generally present to a hospital at a later stage of infection, with clinical symptoms and greater disease severity. Of note, early initiation of antivirals for the treatment of influenza in outpatients has shown significant clinical benefit (28). While there are differences compared with natural infection, the virus challenge model used in our study did result in the development of RSV symptoms, which were evident prior to randomization and continued to increase after treatment in the placebo arm. Our study design enabled a wide range of endpoints to be assessed, establishing the antiviral activity and safety of RV521 in healthy adults.

In conclusion, we demonstrate that therapeutic oral administration of RV521 exceeds target mean trough levels and significantly reduces RSV viral load and clinical symptoms at both 200-mg and 350-mg doses. Furthermore, RV521 is well tolerated, showed no evidence of clinical resistance, and compares favorably to other anti-RSV agents tested in other similar challenge studies. These findings provide sound justification for progression to efficacy studies of RV521 in vulnerable, naturally infected infant and adult target populations.

## MATERIALS AND METHODS

### Study design.

This randomized, double-blind, placebo-controlled trial (ClinicalTrials.gov identifier NCT03258502; EudraCT number 2017-001282-24) was conducted in a purpose-built, specialist viral challenge quarantine unit (hVIVO, London, UK). The study was approved by the North East–Tyne & Wear South Research Ethics Committee, United Kingdom, and was conducted according to Declaration of Helsinki and International Conference on Harmonisation Good Clinical Practice guidelines.

### Subjects.

The study was conducted outside the natural RSV season in healthy male or female adult volunteers aged 18 to 45 years. Only subjects with low serum RSV neutralizing antibody levels (RSV-A Memphis-37b microneutralization antibody titer of ≤810 at screening) were eligible for inclusion, in order to achieve an optimal rate of successful RSV infection after viral challenge. Without such screening for low RSV antibody levels, only around 50% of RSV-A Memphis-37b-challenged adults become infected (29). Infection rates are higher (approximately 75%) after RSV-A Memphis-37 challenge if subjects are selected to have low RSV microneutralization titers (26). Exclusion criteria included a smoking history of ≥10 pack-years; reduced lung function (FEV_1_, <80% of predicted normal); significant nose or nasopharynx abnormalities; symptoms of upper respiratory tract infection or LRTI within the previous 6 weeks; rhinitis (including hay fever); and receipt of medication for hay fever, nasal congestion, or respiratory tract infections within the 7 days before viral challenge. For complete eligibility criteria, see Table S6. All subjects provided written informed consent.

### Randomization and masking.

Subjects were randomly assigned 1:1:1 to oral RV521 350-mg, RV521 200-mg, or placebo groups (see Supplemental Methods). All study staff, the study sponsor, the principal investigator, laboratory evaluators, and subjects were masked to treatment allocation (RV521 versus placebo). Tamper-evident, sealed, subject-specific envelopes were used. The size, weight, and appearance of placebo and RV521 capsules were matched to ensure study masking.

### Dose selection.

Previous RSV challenge studies achieved antiviral efficacy with peripheral blood trough exposure levels over 3× the protein binding-adjusted *in vitro* EC_90_ (12). Based on the phase 1 PK data, this level of exposure was predicted to be achieved on or after the first dose in the majority of subjects with a 200-mg dose and to be met or exceeded on or after the first dose in all subjects with a 350-mg dose. The *in vitro* EC_90_ was determined using a panel of clinical isolates of RSV (Table S7).

### Procedures.

Subjects were screened outside the RSV season for eligibility, including measurement of RSV-A Memphis-37b-specific neutralizing antibody titer, ≤90 days before virus challenge. Eligible subjects were admitted to the quarantine unit on day −2 or day −1 and were inoculated intranasally on day 0 with the challenge virus, RSV-A Memphis-37b (4 log_10_ PFU/ml, given as one 0.4-ml installation per naris), as previously described (26). Nasal wash sampling every 12 h for confirmation of RSV infection by qualitative integrated cycler PCR (12, 19) began on the morning of day 2. Treatment began 12 h (±1 h) after collection of a nasal wash sample confirming RSV infection or on the evening of day 5 if RSV infection remained unconfirmed by the morning of day 5.

The study comprised two consecutive cohorts. Subjects in the first cohort received 200 mg RV521 or placebo; those in the second received 350 mg RV521 or placebo. In each cohort, subjects were assigned 2:1 to RV521 or placebo groups, and therefore the combination of the two placebo groups for analysis resulted in a 1:1:1 overall allocation. In each cohort, subjects received 10 consecutive doses of RV521 or placebo, administered in a fasted state as oral capsules, approximately 12 h apart. Subjects were discharged on day 12 if nasopharyngeal swab samples tested negative by RSV rapid antigen assay (QuickVue RSV test; Quidel, San Diego, CA, USA). If such tests were positive or if a subject remained symptomatic, quarantine was extended to allow further observation. All subjects were evaluated on day 28 (±3 days).

### Assessments.

Twice-daily collection of nasal wash samples allowed measurement of viral load via RT-qPCR and quantitative culture. RT-qPCR results were reported as PFUe/ml when the standard curve included in each assay contained a known infectious amount (PFU) of RSV, as described previously (30). Subjects reported the occurrence and severity of symptoms three times daily using a 10-item subject symptom diary card as previously reported (12) (see Supplemental Methods). Throughout the quarantine period, used tissues were collected and weighed daily, and total nasal mucus weight was recorded. PK assessments were based on venous blood samples. Safety assessments included measurement of vital signs, standard 12-lead ECG recordings, spirometry, a complete physical examination, and a respiratory-directed physical examination. AEs were monitored daily, coded according to the Medical Dictionary for Regulatory Activities Version 20.0, and graded according to the National Cancer Institute Common Terminology Criteria for Adverse Events Version 4.0 (see Supplemental Methods for further details).

To detect any viral variants following RV521 treatment, mutation detection analyses were performed for all subjects with confirmed RSV infection. Nasal wash samples were selected for RSV-F gene sequencing at the time of RT-qPCR viral load peak and at the last time point with an RT-qPCR viral load of >1 log10 PFUe/ml for each subject. All samples were analyzed by population sequencing of the entire RSV-F gene, which was then compared with the inoculation strain RSV-A Memphis-37b challenge virus F gene sequence (27).

### Endpoints.

The primary efficacy endpoint was AUC for RSV viral load, as measured by RT-qPCR, in nasal wash samples taken twice daily from just before the first dose until discharge (day 12). Viral load data were provided as log_10_ PFUe/ml; AUC (log_10_ PFUe/ml · h) was calculated using the trapezoid rule. Secondary efficacy endpoints related to viral load were AUC of RSV viral load, as assessed by quantitative culture, and the following measures, which were each assessed using both RT-qPCR and quantitative culture: peak viral load, time to peak viral load, and time to <1 log_10_ (for RT-qPCR) or undetectable (for quantitative culture) viral load (considered to occur at the first confirmed undetectable assessment after which no further virus was detected). Secondary efficacy endpoints related to clinical symptoms included AUC total symptom score; peak total symptom score; time to peak total symptom score; and the total weight of nasal mucus produced. Other secondary outcomes were safety, PK, and sequence detection of viral variants (see Supplemental Methods).

### Statistical analysis.

Based on the assumption that there would be a 70% reduction in viral load AUC (as measured by RT-qPCR) with RV521 versus placebo during the postinoculation period, it was calculated that 11 subjects in each of the three treatment groups needed to be evaluable for the primary endpoint to achieve 80% power and a two-sided 5% level of significance. However, to account for a lower than expected infection rate and possible dropouts, 22 subjects were to be inoculated and randomized in each of the three treatment groups.

The primary efficacy analysis population (the ITT-I analysis set) comprised all randomized subjects who received challenge virus and at least one dose of study drug and met the criterion for laboratory-confirmed RSV infection (presence of detectable RSV). Sensitivity analyses were based on a subset of the ITT-I population who had first detectable RSV infection prior to study drug administration (the ITT-A analysis set) and a fixed time period of 6.5 days (comprising data for all subjects for 6.5 days of evaluation after the first dose, regardless of the staggered commencement of dosing). The safety analysis set comprised all subjects who received challenge virus; the PK analysis set comprised all subjects who received the challenge virus and provided at least one postdose PK result.

All statistical analyses were performed using two-sided testing. Student’s *t* tests were performed for normally distributed data with a constant variance; otherwise, the Satterthwaite *t* test or nonparametric tests were used. For the mucus weight analysis, the least-squares mean was calculated from a mixed model with repeated measures, adjusted for baseline mucus weight and treatment group as covariates and subject as a random effect.

PK parameters were derived by noncompartmental analysis using Phoenix WinNonlin version 6.4.1. Statistical analyses were performed using SAS software version 9.4 or later.

### Data availability.

The study is registered under EudraCT number 2017-001282-24 (https://www.clinicaltrialsregister.eu/ctr-search/trial/2017-001282-24/results).

## Supplementary Material

Supplemental file 1
