# Supplementary material for: A Randomized, Placebo-Controlled, Respiratory Syncytial Virus Human Challenge Study of the Antiviral Efficacy, Safety, and Pharmacokinetics of RV521, an Inhibitor of the RSV-F Protein
Source: Antimicrob Agents Chemother. 2020 Jan 27;64(2):e01884-19. doi: 10.1128/AAC.01884-19 (PMC6985722; doi:10.1128/AAC.01884-19)
Supplement: Supplemental file 1 [file AAC.01884-19-s0001.pdf]

SUPPLEMENTARY MATERIAL FOR:

Human Antiviral Efficacy, Safety and Pharmacokinetics of RV521, an Inhibitor of the RSV-F Protein: A Randomized, Placebo-Controlled, Respiratory Syncytial Virus Challenge Study

John DeVincenzo MD,[a,b]# Dereck Tait FRCPath,[c] John Efthimiou MD,[d] Julie Mori PhD,[e] Young-In Kim PhD,[a,b] Elaine Thomas PhD,[c] Lynn Wilson PhD,[c] Rachel Harland PhD,[c] Neil Mathews PhD,[c] Stuart Cockerill PhD,[c] Kenneth Powell PhD,[c] Edward Littler PhD[c]

[a]University of Tennessee Center for Health Sciences, Memphis, TN, USA

[b]Children's Foundation Research Institute at LeBonheur Children's Hospital, Memphis, TN, USA

[c]ReViral Ltd, Stevenage, Hertfordshire, UK

[d]Independent Respiratory Specialist, Oxford, UK

[e]hVIVO Services Limited, London, UK

#Address correspondence to John DeVincenzo, jdevince@uthsc.edu

Children's Foundation Research Institute, Le Bonheur Children's Hospital, Room 400R, 50 North Dunlap Street, Memphis, TN 38103

CONTENTS

SUPPLEMENTARY METHODS 3

SUPPLEMENTARY FIGURES AND TABLES 5

FIG S1 Structure of RV521 5

FIG S2 Time to <1 log10 viral load, post-first dose of RV521/placebo, as measured in the nasal wash by RT-qPCR (A)a and time to undetectable viral load, post-first dose of RV521/placebo, as measured in the nasal wash by quantitative culture (B)b (ITT-I analysis set) 6

TABLE S1 Sensitivity analysis of viral load endpoints (ITT-A analysis set) 8

TABLE S2 Sensitivity analysis of disease severity-related endpoints (ITT-A analysis set) 11

TABLE S3 Sensitivity analysis of viral load and clinical symptom-related endpoints based on fixed time period of 6.5 days (ITT-I and ITT-A analysis set) 14

TABLE S4 Overall summary of treatment-emergent adverse events (safety analysis set) 17

TABLE S5 Pharmacokinetic parameters following single and repeated twice-daily dosing of RV521 (pharmacokinetic analysis set) 21

TABLE S6 Full study eligibility criteria 22

TABLE S7 *In vitro* susceptibility of RSV to RV521 27

SUPPLEMENTARY METHODS

Randomization and masking. Subjects were assigned treatment according to a computer-generated randomization schedule. A designated unblinded statistician, who had no further involvement in the conduct or analysis of the study, generated the randomization schedule.

Assessments. Study day 0 was defined as the day of respiratory syncytial virus intranasal exposure. Nasal wash samples were taken twice-daily from study day 2 to 11, and once on the day of discharge (study day 12). Subjects reported the occurrence and severity of symptoms using a 10-item subject symptom diary card, which was completed once on day –1, once on day 12, and three-times daily during the period in between. Nine symptoms (runny nose, stuffy nose, sneezing, sore throat, earache, malaise, cough, headache, muscle ache/joint ache/stiffness) were graded on a scale of 0 to 3, and shortness of breath was graded on a scale of 0 to 4; grade 0 is absence, grade 1 is just noticeable, grade 2 is bothersome but does not prevent participation in activities, grade 3 is bothersome and interferes with activities, and grade 4 is symptom at rest. In order to record total nasal mucus weight, subjects were given pre-weighed packets of paper tissues and asked to place used tissues in a specified bag (sealed to prevent evaporation), which was collected and weighed daily throughout the quarantine period.

Pharmacokinetic (PK) assessments were based on venous blood samples, taken from randomization through to discharge. Safety assessments included measurement of vital signs, standard 12-lead electrocardiogram recordings, and spirometry, each conducted at prespecified timepoints throughout the study period. A complete physical examination and a respiratory-directed physical examination, which included examination of the ears, eyes, nose, throat and chest, was conducted once daily from screening throughout the quarantine period, and at the follow-up visit. Urine samples for determination of urinalysis parameters and blood samples to determine biochemistry and hematology, thyroid function, coagulation, and cardiac enzymes, were taken at prespecified timepoints throughout the study. Adverse events (AEs) were defined as any untoward medical occurrence in subjects, and could, therefore, be any unfavourable event associated with the use of the study drug, whether or not considered related to the study drug, or for the purposes of this study, the challenge virus. Challenge virus-related symptoms recorded in a subject's symptom diary card were expected and presumed to represent virus infection consequent to viral challenge. Typical/normal viral symptoms captured via symptom diary cards were not included as AEs; symptoms considered

unexpected, in the opinion of the investigator, were captured as AEs. Following the viral challenge, upper and lower respiratory tract symptoms identified by the directed physical examination were expected, presumed to represent virus infection consequent to viral challenge and not captured as an AE unless they met the criteria for an AE and were deemed clinically significant in the opinion of the investigator. AEs were monitored daily from screening (day –2) through to the follow-up visit (day 28) and coded to system organ class and preferred term according to the Medical Dictionary for Regulatory Activities Version 20.0. AEs were graded according to the National Cancer Institute Common Terminology Criteria for Adverse Events Version 4.0, and the relationship to the investigational medical product or to challenge virus was recorded. An AE with an onset date/time after administration of the first dose of RV521 is described as a treatment-emergent AE. In this way, AEs occurring after challenge virus inoculation and prior to administration of RV521 that were considered by the investigator to be causally related to challenge virus can be included in the analysis of AEs.

Outcomes. Safety outcomes included the occurrence of AEs. PK outcomes included the maximum plasma concentration, the minimum plasma concentration, the time to maximum plasma concentration, and the area under the plasma concentration-time curve with a dosing interval, calculated by the linear trapezoidal rule.

SUPPLEMENTARY FIGURES AND TABLES

FIG S1 Structure of RV521

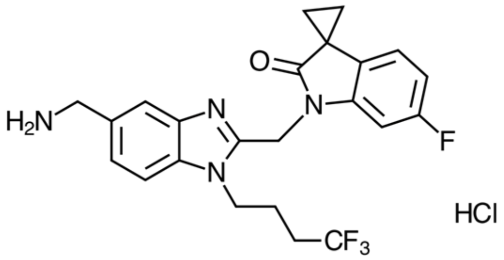

FIG S2 Time to <1 log10 viral load, post-first dose of RV521/placebo, as measured in the nasal wash by RT-qPCR (A)[a] and time to undetectable viral load, post-first dose of RV521/placebo, as measured in the nasal wash by quantitative culture (B)[b] (ITT-I analysis set)

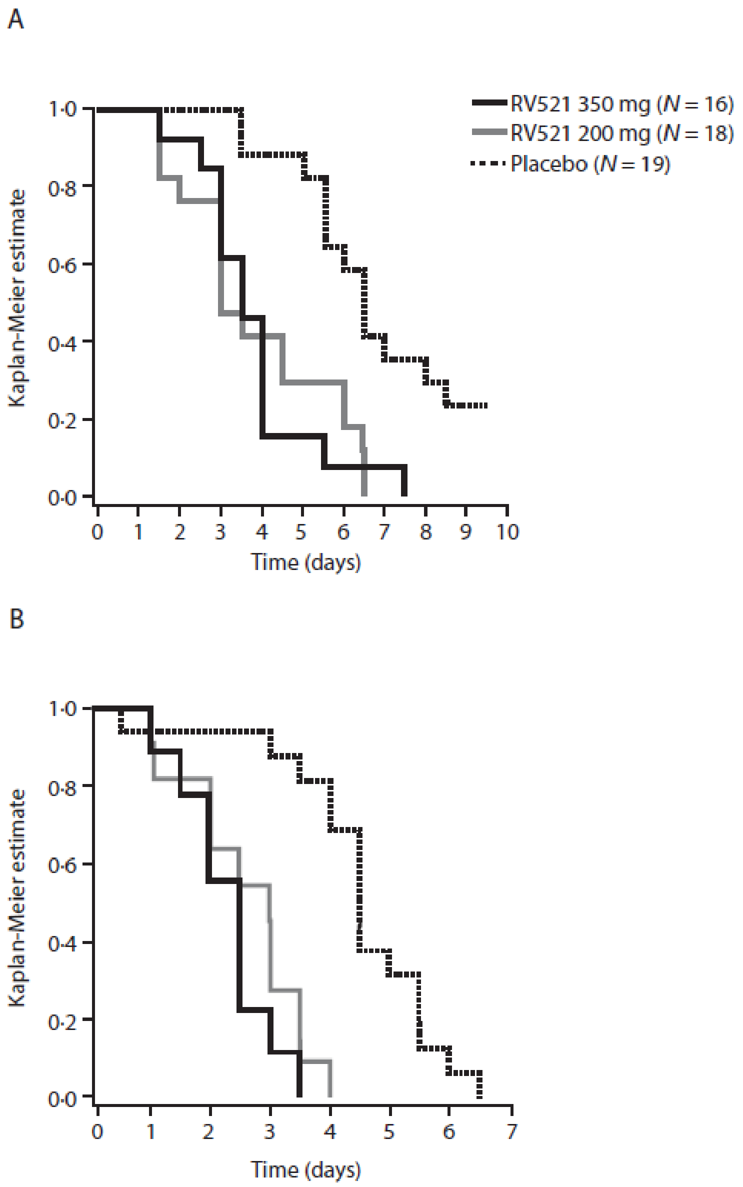

[a]*P*=0.0001 RV521 350 mg versus placebo; *P*=0.0003 RV521 200 mg versus placebo.

[b]*P*<0.0001 both RV521 groups versus placebo.

ITT-I, intent-to-treat infected (defined as all randomized subjects who received the challenge virus and at least one dose of study drug and met the criterion for laboratory-confirmed RSV

infection [presence of viral shedding]); RSV, respiratory syncytial virus; RT-qPCR, reverse transcriptase quantitative PCR.

TABLE S1 Sensitivity analysis of viral load endpoints (ITT-A analysis set)

**Parameter**	**Treatment group**		
	**RV521 350 mg** ***N* = 14**	**RV521 200 mg** ***N* = 17**	**Placebo** ***N* = 16**
AUC of viral load (RT-qPCR), hours x $\log_{10}$ PFUe/mL			
Mean (SE)	210.61 (29.76)	236.81 (37.63)	588.21 (86.50)
Difference in mean relative to placebo (95% CI)	–377.60 (–569.45, –185.77)	–351.40 (–547.85, –154.97)	
Reduction in mean vs placebo (%)	64.19	59.74	
P value[a]	0.0006	0.001	
AUC of viral load (quantitative culture), hours x $\log_{10}$ PFU/mL			
Mean (SE)	43.76 (14.73)	53.89 (15.47)	188.87 (41.63)
Reduction in mean vs placebo (%)	76.83	71.47	
P value[b]	0.01	0.013	
Peak viral load (RT-qPCR), $\log_{10}$ PFUe/mL			
Mean (SE)	3.58 (0.40)	3.63 (0.26)	5.43 (0.37)
Difference in mean relative to placebo (95% CI)	–1.85 (–2.97, –0.73)	–1.8 (–2.72, –0.89)	

P value[c]	0.002	<0.0004	
Peak viral load (quantitative culture), log_{10} PFU/mL			
Mean (SE)	1.81 (0.44)	1.82 (0.41)	3.60 (0.49)
Difference in mean relative to placebo (95% CI)	–1.79 (–3.15, –0.44)	–1.78 (–3.07, –0.50)	
P value[c]	0.011	0.008	
Time to peak viral load (RT-qPCR), days			
Mean (SE)	1.65 (0.39)	0.94 (0.11)	2.62 (0.32)
Difference in mean relative to placebo (95% CI)	–0.98 (–2.01, 0.05)	–1.68 (–2.38, –0.98)	
P value[a]	0.062	<0.0001	
Time to peak viral load (quantitative culture), days			
Mean (SE)	3.47 (0.87)	3.53 (0.84)	3.03 (0.54)
P value[b]	0.603	0.368	
Time to <1 log_{10} viral load, (RT-qPCR), days			
Median (Q1, Q3)	3.5 (3.0, 4.0) (*n* = 13)	3.0 (3.0, 6.0) (*n* = 17)	6.5 (5.5, NE) (*n* = 16)
P value[d]	0.0002	0.0003	
Time to undetectable viral load, (quantitative culture), days[e]			

Median (Q1, Q3)	2.5 (2, 2.5)<br>(*n* = 9)	3 (2, 3.5)<br>(*n* = 11)	4.5 (4, 5.5)<br>(*n* = 14)
P value[d]	<0.0001	<0.0001	

[a]Satterthwaite test.

[b]Wilcoxon rank-sum test.

[c]t-test.

[d]Kaplan–Meier log-rank test.

[e]Time from the peak until the first undetectable viral load after which no further detectable viral load occurred.

AUC, area under the curve; CI, confidence interval; ITT-A, intent-to-treat infected A (defined as all randomized subjects who received the challenge virus and at least one dose of study drug and met the criterion for laboratory-confirmed RSV infection [presence of viral shedding] prior to administration of study drug); NE, not estimable; PFU(e), plaque forming unit (equivalents); RSV, respiratory syncytial virus; RT-qPCR, reverse transcriptase quantitative PCR; SE, standard error.

TABLE S2 Sensitivity analysis of disease severity-related endpoints (ITT-A analysis set)

**Parameter**	**Treatment group**		
	**RV521 350 mg** ***N* = 14**	**RV521 200 mg** ***N* = 17**	**Placebo** ***N* = 16**
AUC of total symptom score (score x hours)			
Mean (SE)	92.98 (26.81)	117.9 (35.26)	437.62 (127.87)
Reduction in mean vs placebo (%)	78.75	73.06	
P value[a]	0.002	0.005	
Peak total symptom score			
Mean (SE)	2.1 (0.49)	2.5 (0.49)	5.7 (1.26)
Difference in mean relative to placebo (95% CI)	–3.54 (–6.37, –0.72)	–3.22 (–6.03, –0.40)	
P value[b]	0.016	0.027	
Time to peak total symptom score, days			
Mean (SE)	1.26 (0.55)	1.82 (0.67)	2.08 (0.227)
Difference in mean relative to placebo (95% CI)	–0.82 (–2.06, 0.43)	–0.25 (–1.73, 1.22)	
P value[b]	0.184	0.726	

Time to resolution from peak total symptom score, days[c]			
Mean (SE)	2.44 (0.43) (n = 13)	2.73 (0.39) (n = 14)	4.19 (0.54) (n = 16)
Difference in mean relative to placebo (95% CI)	–1.75 (–3.21, –0.28)	–1.46 (–2.85, –0.06)	
P value[d]	0.021	0.042	
Daily nasal mucus weight, g			
LS mean[e]	0.26	0.32	0.68
Difference in LS mean relative to placebo (%)	61.76	52.94	
P value[a]	0.010	0.02	

[a]Wilcoxon rank-sum test.

[b]Satterthwaite test.

[c]Time from peak symptoms until the start of the first 24-hour symptom-free period after the peak.

[d]t-test.

[e]LS mean was calculated from a mixed model with repeated measures, adjusted for baseline mucus weight and treatment group as covariates, and subject as a random effect. The *P* value represents the LS mean difference between treatment groups.

AUC, area under the curve; CI, confidence interval; ITT-A, intent-to-treat infected A (defined as all randomized subjects who received the challenge virus and at least one dose of study drug and met the criterion for laboratory-confirmed RSV infection [presence of viral

shedding] prior to administration of study drug); LS, least squares; RSV, respiratory syncytial virus; SE, standard error.

TABLE S3 Sensitivity analysis of viral load and clinical symptom-related endpoints based on fixed time period of 6.5 days (ITT-I and ITT-A analysis set)

**Parameter**	**Treatment group**		
	**RV521 350 mg**	**RV521 200 mg**	**Placebo**
**ITT-I population**	***N* = 16**	***N* = 18**	***N* = 19**
AUC of viral load (RT-qPCR), log_{10} PFUe/mL x hours			
Mean (SE)	182.59 (30.29)	221.98 (37.05)	435.96 (65.12)
Reduction in mean vs placebo (95% CI)	–253.37 (–401.21, –105.53)	–213.98 (–367.35, –60.61)	
Reduction in mean vs placebo (%)	58.12	49.08	
P value[a]	0.002	0.008	
AUC of viral load (quantitative culture), log_{10} PFU/mL x hours			
Mean (SE)	38.29 (13.36)	50.98 (14.89)	162.35 (37.77)
Reduction in mean vs placebo (%)	76.42	68.60	
P value[b]	0.012	0.027	
AUC of total symptom score (score x hours)			
Mean (SE)	82.41 (24.45)	111.35 (33.88)	381.82 (111.59)
P value[b]	0.003	0.009	

**ITT-A population**	***N* = 14**	***N* = 17**	***N* = 16**
AUC of viral load (RT-qPCR), $\log_{10}$ PFUe/mL x hours			
Mean (SE)	207.56 (28.74)	234.29 (37.07)	510.51 (60.66)
Reduction in mean vs placebo (95% CI)	–302.95 (–442.44, –163.46)	–276.21 (–422.62, –129.81)	
P value[a]	0.0002	0.0007	
AUC of viral load (quantitative culture), $\log_{10}$ PFU/mL x hours			
Mean (SE)	43.76 (14.73)	53.98 (15.47)	188.87 (41.63)
Reduction in mean vs placebo (%)	76.83	71.42	
P value[b]	0.01	0.013	
AUC of total symptom score (score x hours)			
Mean (SE)	92.98 (26.81)	117.41 (35.26)	397.81 (114.04)
P value[b]	0.003	0.005	

[a]Satterthwaite test.

[b]Wilcoxon rank-sum test.

AUC, area under the curve; CI, confidence interval; ITT-A, intent-to-treat infected A (defined as all randomized subjects who received the challenge virus and at least one dose of study drug and met the criterion for laboratory-confirmed RSV infection [presence of viral shedding] prior to administration of study drug); ITT-I, intent-to-treat infected (defined as all randomized subjects who received challenge virus and at least one dose of study drug, and met the criterion for laboratory-confirmed RSV infection [presence of viral shedding]);

PFU(e), plaque forming unit (equivalents); RSV, respiratory syncytial virus; RT-qPCR, reverse transcriptase quantitative PCR; SE, standard error.

TABLE S4 Overall summary of treatment-emergent adverse events (safety analysis set)

	**Treatment group**		
**TEAE, number of subjects (%)**	**RV521 350 mg** **$N = 22$**	**RV521 200 mg** **$N = 22$**	**Placebo** **$N = 22$**
Total	16 (73)	11 (50)	11 (50)
**Blood and lymphatic system disorders**	**0**	**0**	**1 (5)**
Lymphadenopathy	0	0	1 (5)
**Cardiac disorders**	**0**	**0**	**1 (5)**
Myocarditis	0	0	1 (5)
**Eye disorders**	**0**	**0**	**2 (9)**
Eye pain	0	0	1 (5)
Ocular hyperemia	0	0	1 (5)
**Gastrointestinal disorders**[a]	**13 (59)**	**7 (32)**	**2 (9)**
Abdominal discomfort	1 (5)	0	0
Abdominal distention	1 (5)	0	0
Abdominal pain	4 (18)	2 (9)	0
Abdominal pain lower	1 (5)	0	0
Diarrhoea	9 (41)	3 (14)	1 (5)
Food poisoning	0	1 (5)	1 (5)

Lip dry	1 (5)	0	0
Nausea	12 (55)	2 (9)	2 (9)
Vomiting	2 (9)	1 (5)	0
**General disorders and administration site conditions**	**1 (5)**	**1 (5)**	**3 (14)**
Catheter site erythema	1 (5)	0	0
Catheter site paraesthesia	0	1 (5)	1 (5)
Pyrexia	0	0	1 (5)
Vessel puncture site erythema	0	0	1 (5)
**Infections and infestations**	**5 (23)**	**3 (14)**	**1 (5)**
Gastroenteritis	1 (5)	0	0
Rhinitis	2 (9)	1 (5)	1 (5)
URTI	0	2 (9)	0
Viral URTI	2 (9)	0	0
**Injury, poisoning and procedural complications**	**0**	**1 (5)**	**1 (5)**
Joint injury	0	0	1 (5)
Skin abrasion	0	1 (5)	0

**Metabolism and nutrition disorders**	**0**	**1 (5)**	**0**
Decreased appetite	0	1 (5)	0
**Musculoskeletal and connective tissue disorders**	**1 (5)**	**0**	**3 (14)**
Musculoskeletal pain	0	0	1 (5)
Myalgia	1 (5)	0	1 (5)
Pain in jaw	0	0	1 (5)
**Nervous system disorders**	**1 (5)**	**2 (9)**	**4 (18)**
Dizziness	1 (5)	1 (5)	1 (5)
Headache	0	0	2 (9)
Hypoaesthesia	0	1 (5)	0
Tension headache	0	0	1 (5)
**Respiratory, thoracic and mediastinal disorders**	**0**	**1 (5)**	**1 (5)**
Epistaxis	0	1 (5)	0
Oropharyngeal pain	0	0	1 (5)
**Skin and subcutaneous tissue disorders**	**1 (5)**	**1 (5)**	**2 (9)**

Dry skin	1 (5)	1 (5)	0
Rash	0	0	2 (9)

Safety analysis set comprised all subjects who received the challenge virus, regardless of whether they received study drug.

[a]All study drug dosing was in the fasted state.

TEAE, treatment-emergent adverse event; URTI, upper respiratory tract infection.

TABLE S5 Pharmacokinetic parameters following single and repeated twice-daily dosing of RV521 (pharmacokinetic analysis set)

	**Treatment group**			
	**RV521 350 mg**		**RV521 200 mg**	
**Parameter**	**Dose 1 (*N* = 22)**	**Dose 10 (*N* = 21)**	**Dose 1 (*N* = 22)**	**Dose 10 (*N* = 22)**
Mean C_{max} (SD), ng/mL	169 (68.4)	294 (194)	55.3 (35.7)	94.9 (49.0)
Mean C_{min} (SD), ng/mL	80.0 (48.7)	151 (125)	22.6 (16.3)	39.4 (19.1)
Median T_{max} (range), hours	6.00 (2.00–8.00)	5.05 (2.60–7.22)	5.04 (4.00–8.00)	5.06 (0–6.98)
Geometric mean (CV) $AUC_{0–12h}$, ng.h/mL	953 (54.2)	1900 (94.2)	255 (125)	642 (61.7)
Geometric mean (CV) $T_{1/2}$, hours		9.35 (13.3)		8.54 (117.4)

The PK analysis set comprised all randomized subjects who received the challenge virus and at least one dose of study drug, and provided at least one post-dose PK result.

$AUC_{0–12h}$, area under the plasma concentration-time curve with a dosing interval, calculated by the linear trapezoidal rule; C_{max}, maximum plasma concentration; C_{min}, minimum plasma concentration with a dosing interval (equivalent to the observed concentration at 12 hours post-dose); CV, coefficient of variation; PK, pharmacokinetic; SD, standard deviation; $T_{1/2}$, apparent terminal half-life; T_{max}, time at which the maximum plasma concentration occurs.

TABLE S6 Full study eligibility criteria

**Inclusion criteria**
Aged 18–45 years
In good health with no history of major medical conditions that would interfere with subject safety, as defined by medical history, physical examination, and routine laboratory tests, as determined by the investigator at a screening evaluation
A documented medical history either prior to entering the study and/or following medical history review with the study physician at screening
A total body weight ≥50 kg and BMI ≥18 kg/m^2 and ≤30 kg/m^2
The following inclusion criteria are applicable to subjects undergoing viral challenge who are in a sexual relationship:<br>• Female subjects must have a negative pregnancy test at screening and just prior to the date of viral challenge<br>• Female subjects of childbearing potential must be using contraception consisting of two forms of birth control (one of which must be a barrier method) starting from at least 2 weeks prior to entry to quarantine and continuing until 90 days after the date of viral challenge/last dosing with IMP (whichever occurs last)<br>• Female subjects who are no longer of child bearing potential<br>• Male subjects must agree to contraceptive requirements at entry to quarantine, and continuing until 90 days after the date of viral challenge/last dosing with IMP (whichever occurs last)
An informed consent document signed and dated by the subject and the investigator or designee

Sero-suitable to the challenge virus (the serology result obtained suggests that the subject is sensitive to RSV infection, i.e. they are likely to be infected following inoculation with the challenge virus)
A history of childhood asthma before the age of 12 years is acceptable provided the subject is asymptomatic without treatment. Subjects who have experienced ≤1 mild episode of wheeze (mild is defined as having been treated with bronchodilators only), may be included at the investigator's discretion, providing the episode lasted ≤2 weeks, and ended >1 year ago
**Exclusion criteria**
Subjects who have smoked >10 pack-years at any time<br>• In subjects who have smoked <10 pack-years at any time, use of tobacco or nicotine-containing products in any form, in the last month prior to admission
Females who are breastfeeding, or have been pregnant within 6 months prior to the study, or have a positive pregnancy test at any point during screening or prior to viral challenge
Subjects who have:<br>• Any history or evidence of any clinically significant or currently active CV, respiratory, dermatological, GI, endocrinological, hematological, hepatic, immunological (including immune suppression), metabolic, urological, renal, neurological or psychiatric disease)<br>• And/or other major disease, including a history of malignancy, that, in the opinion of the investigator, may interfere with a subject completing the study and the necessary investigations
FEV_1 <80%
Any history of physician diagnosed and/or objective test confirmed asthma, COPD, pulmonary hypertension, or chronic lung condition of any aetiology (see inclusion criteria above regarding asthma)

Positive HIV, active HAV, HBV or HCV
Any significant abnormality altering the anatomy of the nose or nasopharynx in a substantial way that may interfere with the aims of the study and in particular any of the nasal assessments or viral challenge
Any clinically significant history of epistaxis within the last 3 months and/or history of being hospitalized due to epistaxis on any previous occasion
Any nasal or sinus surgery within 3 months of the date of viral challenge
Twelve-lead ECG recording with clinically relevant abnormalities as judged by the study physician/PI
Confirmed positive test for drugs of abuse or cotinine on admission
Venous access deemed inadequate for the phlebotomy and cannulation demands of the study
Presence of fever, defined as subject presenting with a temperature reading of ≥ 37.9°C on day –2, day –1 and/or pre-challenge on day 0
Evidence of vaccinations within the 4 weeks prior to the planned date of viral challenge<br>• Intention to receive any vaccination(s) before the follow-up visit (day 28)
Those employed or immediate relatives of those employed at hVIVO or the Sponsor
Receipt of blood or blood products, or blood loss (including blood donations) of ≥470 mL during the 3 months prior to the planned date of viral challenge or planned during the 3 months after the final visit
Prior/concurrent medication:<br>• Use within 7 days prior to the planned date of viral challenge of any medication or product (prescription or OTC) for symptoms of hay fever, dermatitis, nasal congestion or respiratory tract infections including the use of regular nasal or dermal corticosteroids or antibiotics

• Use within 7 days prior to day –2/day –1 of short and long-acting antihistamines and nasal mast cell stabilisers<br>• History of immunotherapy or concurrently undergoing immunotherapy treatment<br>• Receipt of any systemic chemotherapy agent, immunoglobulins, or any other cytotoxic or immunosuppressive drugs at any time
Participation in previous studies:<br>• Receipt of any IMP within 3 months prior to the planned date of viral challenge<br>• Receipt of >3 IMPs within the previous 12 months prior to the planned date of viral challenge<br>• Prior inoculation with a virus from the same virus-family as the challenge virus<br>• Prior participation in another HVC study with a respiratory virus in the preceding 12 months taken from the date of viral challenge/first dosing with IMP (whichever occurs first) in the previous study to the date of expected viral challenge in this study
Receipt of systemic (intravenous and/or oral) glucocorticoids or systemic antiviral drugs within 3 months prior to the planned date of viral challenge
History or currently active symptoms suggestive of URTI or LRTI within 6 weeks prior to viral challenge
Use or anticipated use during the conduct of the study of concomitant medications (prescription and/or OTC), including vitamins or herbal and dietary supplements within the specified windows, unless in the opinion of the PI, the medication will not interfere with the study procedures or compromise subject safety
Positive pregnancy test at any point during screening or prior to viral challenge
History of anaphylaxis and/or a history of severe allergic reaction or significant intolerance to any food or drug, as assessed by the PI

History or presence of alcohol addiction, or excessive use of alcohol, or excessive consumption of xanthine containing substances
Any other finding that, in the opinion of the investigator, deems the subject unsuitable for the study
Subjects of Japanese ethnicity, as determined by the following criteria being met; subjects must have lived outside of Japan for ≤5 years in total and be first generation Japanese, defined as born in Japan and having four biologic grandparents who are ethnic Japanese

BMI, body mass index; COPD, chronic obstructive pulmonary disease; CV, cardiovascular; ECG, electrocardiogram; FEV_1, forced expiratory volume in 1 second; GI, gastrointestinal; HAV, hepatitis A; HBV, hepatitis B; HCV, hepatitis C; HIV, human immunodeficiency virus; HVC, human viral challenge; IMP, investigational medicinal product; LRTI, lower respiratory tract infection; OTC, over-the-counter; PI, principal investigator; RSV, respiratory syncytial virus; URTI, upper respiratory tract infection.

TABLE S7 *In vitro* susceptibility of RSV to RV521

	**EC_{50} range (nM)**	**EC_{90} range (nM)**
RSV-A (panel of 10 clinical strains)	0.3–1.0	2.5–5.6
RSV-B (panel of 10 clinical strains)	0.4–1.4	2.0–10.4

EC_{50} and EC_{90} were determined by plaque assay.

EC_{50}, 50% effective concentration; EC_{90}, 90% effective concentration; RSV, respiratory syncytial virus.
